# Epidemiologic Evidence for Airborne Transmission of SARS-CoV-2 during Church Singing, Australia, 2020

**DOI:** 10.3201/eid2706.210465

**Published:** 2021-06

**Authors:** Anthea L. Katelaris, Jessica Wells, Penelope Clark, Sophie Norton, Rebecca Rockett, Alicia Arnott, Vitali Sintchenko, Stephen Corbett, Shopna K. Bag

**Affiliations:** Western Sydney Local Health District, Sydney, New South Wales, Australia (A. Katelaris, J. Wells, P. Clark, S. Norton, S. Corbett, S.K. Bag);; The University of Sydney, Sydney (S. Norton, R. Rockett, A. Arnott, V. Sintchenko, S Corbett, S.K. Bag);; New South Wales Health Pathology, Westmead, New South Wales, Australia (R. Rockett, A. Arnott, V. Sintchenko)

**Keywords:** coronavirus disease, COVID-19, severe acute respiratory syndrome coronavirus 2, SARS-CoV-2, coronaviruses, viruses, disease outbreak, infectious disease transmission, presymptomatic, public health practice, church singing, respiratory infections, whole-genome sequencing, epidemiology, zoonoses, Australia

## Abstract

An outbreak of severe acute respiratory syndrome coronavirus 2 infection occurred among church attendees after an infectious chorister sang at multiple services. We detected 12 secondary case-patients. Video recordings of the services showed that case-patients were seated in the same section, up to 15 m from the primary case-patient, without close physical contact, suggesting airborne transmission.

The circumstances under which airborne transmission of severe acute respiratory syndrome coronavirus 2 (SARS-CoV-2) might occur are uncertain (*1*,*2*). Previous cluster reports have suggested involvement of airborne transmission (*3*,*4*), but clear epidemiologic evidence is lacking. We investigated a SARS-CoV-2 outbreak in a church in Sydney, New South Wales, Australia, and reviewed the epidemiologic and environmental findings to assess the possibility of airborne transmission of SARS-CoV-2.

## The Study

On July 18, 2020, the Western Sydney Public Health Unit was notified of a positive SARS-COV-2 test result for an 18-year-old man (PCR cycle threshold [C_t_] values: envelope gene 14.5, nucleocapsid gene 16.8). He had sought testing the day before, after learning of a SARS-COV-2 exposure at a venue he attended on July 11. He reported symptom onset of malaise and headache on July 16 and cough and fever on July 17. He was a church chorist and, during his infectious period (from 48 hours before onset), had sung at four 1-hour services, 1 each on July 15 and 16 and 2 on July 17.

The case-patient had sung from a choir loft, elevated 3.5 m above the congregation, which he entered before and left after the service. He denied touching objects in the church or mixing with the general congregation. Video recordings of the services corroborated this history. We identified close contacts according to the national coronavirus disease (COVID-19) control guidelines at the time (*5*): anyone who had spent >15 min face-to-face or shared a closed space for 2 hours with a case-patient during the infectious period of the case-patient. Initially, 10 other chorists and staff were classified as close contacts and required to quarantine (*5*).

On July 18, the church informed the community about the case-patient, prompting testing among members. On July 20, the Western Sydney Public Health Unit was notified of 2 additional case-patients who reported attendance on July 15 and 16. Neither was known by the primary case-patient.

Because transmission was deemed likely to have occurred at these services, we classified all attendees of the 4 services as close contacts, required to quarantine, and requested to seek baseline SARS-CoV-2 testing regardless of symptoms (in addition to if symptoms developed). Public health staff telephoned attendees (identified by mandatory service sign-in records), released alerts through the church and media, and established a testing clinic on-site. Close contacts were contacted every 2–3 days to inquire about symptoms and advised to retest if symptoms developed.

We identified 508 close contacts across the 4 services (Table), of which 434 (85%) were recorded as having a test within 17 days after exposure. Most contacts were tested 2–7 days after exposure (Appendix Figure 1).

**Table:** Number of SARS-CoV-2 close contacts and case-patients in an outbreak in a church, by service date, Australia, 2020*

Date of service, July	No. contacts†	No. tested‡	Proportion tested, %	No. cases	Secondary attack rate, %
15	215	169	79	5	2.3
16	120	108	90	7§	5.8
17 (2 services)	173	157	91	(1§)	NC
Total	508	434	85	12	2.4

*SARS-CoV-2, severe acute respiratory syndrome coronavirus 2; NC, not calculated. †Contacts identified through church service sign-in records and staff lists. This procedure might slightly underestimate the number of contacts because some persons might not have signed in and some telephone numbers were illegible or invalid.  ‡Contacts were tested within 17 d (14-d incubation period plus 3 d) of the last exposure date. Pathology providers in New South Wales, Australia, routinely report SARS-CoV-2 test results (positive or negative) to public health authorities. This number would not include tests performed under a different name or spelling to that on the sign-in records. §One case-patient attended 2 services on July 16 and 17. Because of the absence of additional case-patients on July 17, we have attributed exposure of this case-patient to have been on July 16.

**Figure 1:**
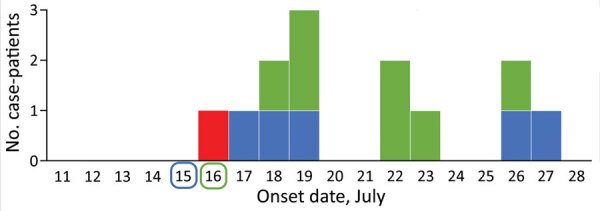
Epidemiologic curve of an outbreak of infection with severe acute respiratory syndrome coronavirus 2 in a church, Australia, 2020. Red indicates symptom onset date for the index case-patient, who sang at 4 services on July 15–17; secondary case-patient symptom onset dates are color coded by date of service attendance as indicated along baseline (1 secondary case-patient attended services on July 16 and 17). The 5 case-patients with onsets of July 22–26 also had exposures to earlier outbreak case-patients in their households.

We detected 12 secondary case-patients among 508 service attendees, yielding an overall secondary attack rate (SAR) of 2.4% across the 4 services (Table). Five case-patients attended only the service on July 15 (SAR 5/215, 2.3%), and 7 attended only on July 16 (SAR 7/120, 5.8%). One case-patient who attended on July 16 also attended on July 17; however, no case-patients were identified who attended only a service on July 17. Secondary case-patients showed development of symptoms 2–12 days after exposure (Figure 1). Five of the secondary case-patients were from the same households as earlier cluster case-patients. Thus, these case-patients might have been infected within the household rather than the church. No secondary case-patients reported other SARS-COV-2 exposures outside these services. There were no deaths, although 3 case-patients were hospitalized, including 2 who required intensive care.

SARS-CoV-2 genome sequencing was performed for the primary case-patient and 10 secondary case-patients (*6*). These case-patients formed a single genomic cluster with a maximum of 2 nt changes from the SARS-CoV-2 genome of the primary case-patient (Appendix Figure 2). High C_t_ values for the remaining 2 case-patients prohibited sequencing.

To further characterize exposures, we determined the seating positions of secondary case-patients within the church. We asked case-patients to describe where they sat, and the video recordings of the services were reviewed, jointly with the case-patients where possible, to confirm locations.

**Figure 2:**
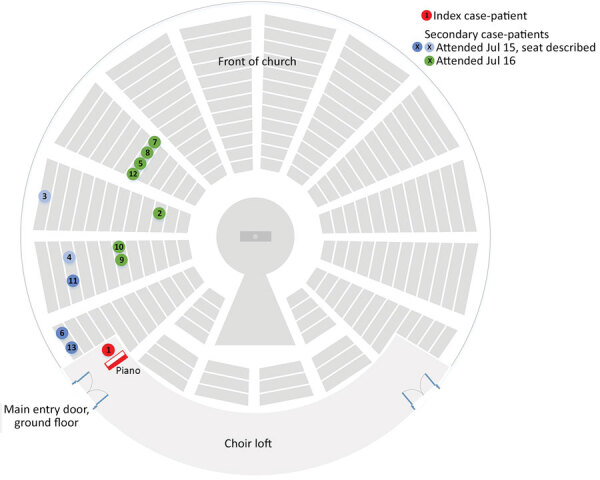
Schematic diagram of church layout showing seating locations of primary and secondary case-patients during an outbreak of infection with severe acute respiratory syndrome coronavirus 2, Australia, 2020. Case numbers are based on order of notification received by the Public Health Unit. Location of case-patients indicated in green and dark blue were confirmed on video recordings; the 2 case-patients indicated in light blue described their locations. The primary case-patient was located in an elevated loft ≈3 m above ground level. He was singing and playing the piano throughout the services and faced toward the piano. Other members of the congregation were seated throughout all sections of the church during the 4 services. Relatively more persons were seated in the front area of the church than in the sides or back.

The church was round, and pews were located circumferentially. We were able to locate the exact location of 10 of the 12 secondary case-patients by using the recordings. The remaining 2 case-patients (case-patients 3 and 4) were unable to review the recordings but described the section and row in which they sat. All secondary case-patients sat within a 70° section, below and 1–15 m from the primary case-patient (Figure 2). The primary case-patient faced away from this area, and used a microphone. Cases were not detected in attendees seated in other sections, and the spatial clustering remains if the 5 potentially household-acquired case-patients are excluded (case-patients 7, 8, 10, 12, and 13). None of the other choristers showed symptoms or tested positive for SARS-CoV-2. Use of masks was not in place.

To understand the ventilation, we conducted 2 site visits with the building manager. The church had a high conical roof, and the ventilation system at the apex was not operating during the services. The doors and windows were largely closed, except as persons entered and exited, and the wall fans were off, meaning there was minimal ventilation.

## Conclusions

We detected 12 secondary case-patients linked to an infectious case-patient at church services on 2 days. Secondary case-patients were seated in the same area of the church, up to 15 m from the primary case-patient, with whom there was no evidence of close physical contact. We believe that transmission during this outbreak is best explained by airborne spread, potentially the result of 3 factors. First, singing has been demonstrated to generate more respiratory aerosol particles and droplets than talking (*7*). Second, minimal ventilation might have enabled respiratory particles to accumulate in the air, and convection currents might have carried particles toward the pews where secondary case-patients were seated. Third, the primary case-patient was likely near the peak of infectiousness on the basis of low C_t_ values (*8*) and symptom onset occurring around the exposure dates (*9*). Although we cannot completely exclude fomite transmission, this transmission would not explain the spatial clustering of case-patients within the church over 2 days.

Strengths of our investigation include detailed case and contact follow-up, availability of video recordings of the services to confirm movements and locations of case-patients, high uptake of testing by contacts, and that SARS-CoV-2 genome sequencing provided supportive evidence that case-patients were closely related genomically. In addition, the New South Wales context of low community transmission (*10*) and high estimated case ascertainment (*11*) makes it unlikely that case-patients acquired infection outside this cluster.

A limitation was that most contacts were tested within a week of exposure, which could have been too early to detect some asymptomatic infections. Second, this investigation only provides circumstantial evidence of airborne transmission, and does not help elucidate the exact mechanism of spread. Finally, we are unsure why transmission did not occur at the services on July 17 (except in 1 possible instance); reasons might be related to altered air flow, the primary case-patient being past peak infectiousness, or that cases that did occur went undetected.

This cluster occurred despite adherence to guidelines requiring microphone use and a 3-m cordon around singers. Guidelines for places of worship were tightened after this cluster was detected, including increasing the distance required around a singer to 5 m. However additional mitigation measures might be necessary to prevent airborne infection during church services and singing, including increased natural or artificial ventilation (*12*) or moving activities outdoors.

Appendix Additional information on airborne transmission of severe acute respiratory syndrome coronavirus 2 during church singing, Australia, 2020.
