# Supplementary material for: Epidemiologic Evidence for Airborne Transmission of SARS-CoV-2 during Church Singing, Australia, 2020
Source: Emerg Infect Dis. 2021 Jun;27(6):1677–80. doi: 10.3201/eid2706.210465 (PMC8153858; doi:10.3201/eid2706.210465)
Supplement: Appendix — Additional information on airborne transmission of severe acute respiratory syndrome coronavirus 2 during church singing, Australia, 2020. [file 21-0465-Techapp-s1.pdf]

Article DOI: https://doi.org/10.3201/eid2706.210465

Epidemiologic Evidence for Airborne Transmission of SARS-CoV-2 during Church Singing, Australia, 2020

Appendix

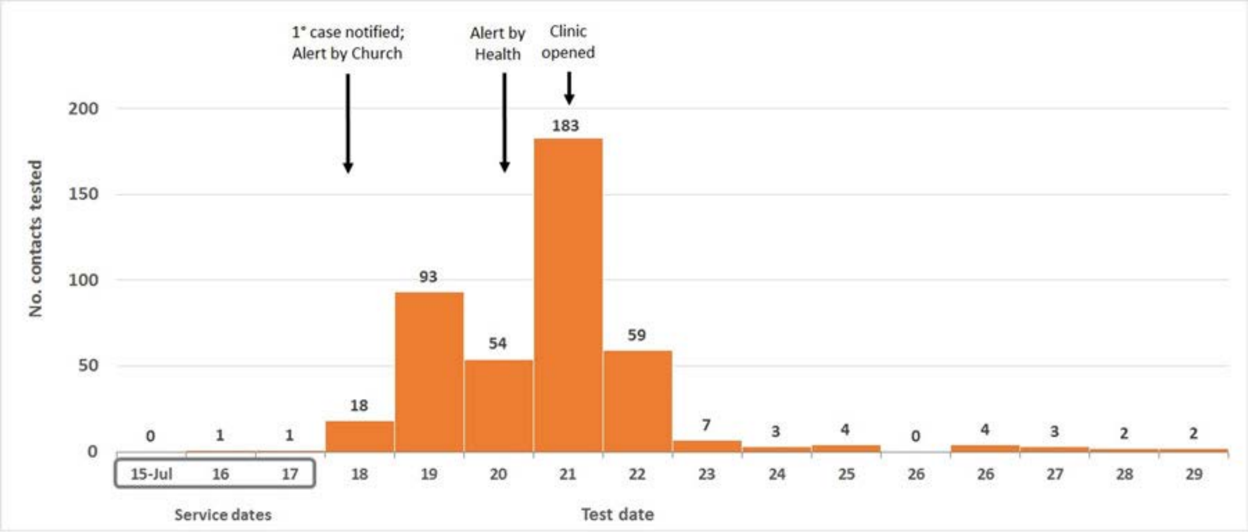

Appendix Figure 1. Number of close contacts tested for severe acute respiratory syndrome coronavirus 2, by date of test, in relation to key events during outbreak in the church, Australia, 2020.

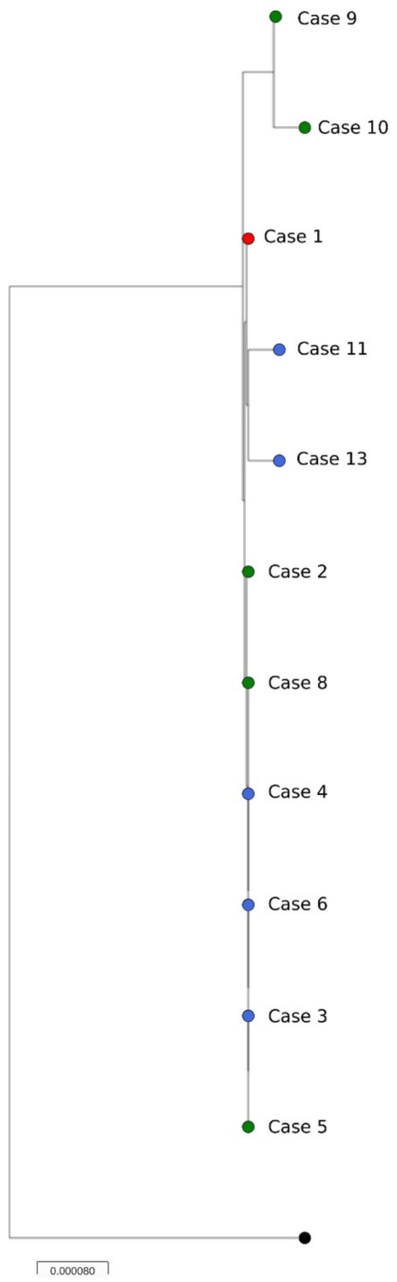

Appendix Figure 2. Phylogenetic analysis of severe acute respiratory syndrome coronavirus 2 (SARS-CoV-2) genomes sequenced from case-patients during outbreak in the church, Australia, 2020. Black node indicates the tree root (GISAID accession no. EPI-ISL-413856); red node indicates primary case-patient; blue node indicates secondary case-patient from July 15 service; and green node indicates secondary case-patient from July 16 service. Scale bar indicates nucleotide substitutions per site. GISAID virus names of SARS-CoV-2 genomes hCoV-19/Australia/NSW657/2020, hCoV-19/Australia/NSW682/2020, hCoV-19/Australia/NSW698/2020, hCoV-19/Australia/NSW699/2020, hCoV-19/Australia/NSW707/2020, hCoV-19/Australia/NSW708/2020, hCoV-19/Australia/NSW715/2020, hCoV-19/Australia/NSW727/2020, hCoV-19/Australia/NSW747/2020, hCoV-19/Australia/NSW755/2020.
